# 

**Published:** 1878-01

**Authors:** 


					﻿Vol. 25.
JANUARY, 1878.
No. 1.
TWENTY-FIFTH YEAR.
HALL’S
Journal of Health.
PUBLISHED AT BIBLE HOUSE,
THIRD AVENUE, CORNER OF NINTH STREET,
TSTZEFW YORK.
E. H. GIBBS, M.D., Editor.
AGENTS FOR THE TRADE’.
AMERICAN NEWS COMPANY, 121 NASSAU STREET.
NEW YORK NEWS COMPANY, 18 BEEKMAN STREET.
Terms, $1.50 a Year, or $1.00 for Eight Months. Postage paid.
SINGLE NUMBER, IB CENTS.
Couch & Johnston. Printers, 11 Frankfort St., New York.
				

